# Identifying Frail Patients by Using Electronic Health Records in Primary Care: Current Status and Future Directions

**DOI:** 10.3389/fpubh.2022.901068

**Published:** 2022-06-22

**Authors:** Jianzhao Luo, Xiaoyang Liao, Chuan Zou, Qian Zhao, Yi Yao, Xiang Fang, John Spicer

**Affiliations:** ^1^International Medical Centre/Ward of General Practice and National Clinical Research Centre for Geriatrics, West China Hospital, Sichuan University, Chengdu, China; ^2^Department of General Practice, Chengdu Fifth People's Hospital, Chengdu University of Traditional Chinese Medicine, Chengdu, China; ^3^GP and Senior Lecturer in Medical Law and Clinical Ethics, Institute of Medical and Biomedical Education, St George's University of London, London, United Kingdom

**Keywords:** frailty, assessment, EHRs, primary care, electronic frailty index

## Abstract

With the rapidly aging population, frailty, characterized by an increased risk of adverse outcomes, has become a major public health problem globally. Several frailty guidelines or consensuses recommend screening for frailty, especially in primary care settings. However, most of the frailty assessment tools are based on questionnaires or physical examinations, adding to the clinical workload, which is the major obstacle to converting frailty research into clinical practice. Medical data naturally generated by routine clinical work containing frailty indicators are stored in electronic health records (EHRs) (also called electronic health record (EHR) data), which provide resources and possibilities for frailty assessment. We reviewed several frailty assessment tools based on primary care EHRs and summarized the features and novel usage of these tools, as well as challenges and trends. Further research is needed to develop and validate frailty assessment tools based on EHRs in primary care in other parts of the world.

## Introduction

In the context of an aging population, frailty is a major public health problem globally (1). Frailty is defined as a state of increased vulnerability to stressors, at the core of which is a decline in the physiological reserves, or even decompensation of multiple organ systems, leading to an increased risk of adverse outcomes (2, 3). This might include falls, hospitalisations, long-term care, disabilities, and mortality (2, 3). A systematic review involving over 60,000 community-dwelling older adults from the US, Canada, Australia, China, and some European countries showed that the average prevalence of frailty was 10.7%, and this percentage nearly tripled among those older than 85 years (4).

It is possible to reverse frailty through intervention (1, 5), and the early identification of patients with frailty is a prerequisite, especially using validated frailty assessment tools (6). Two typical models for frailty assessment that were initially proposed in 2001 are Fried's frailty phenotype and the frailty index (7, 8). The frailty phenotype evaluates whether patients have physical frailty through five clinical presentations, including low grip strength, slow gait speed, unintended weight loss, low physical activity, and self-reported exhaustion (7). The frailty index is based on the cumulative age-related health deficits model proposed by Rockwood and Mitnitski (8). Health deficits are defined in this model as a broad range of health problems that increase with age, such as diseases, symptoms, signs, disability, and abnormal laboratory results, and over 30 deficits are enough for assessment (8, 9). The frailty index score can be calculated by dividing the number of health deficits a patient has by the total number of health deficits (8, 9). Based on the theory of the cumulative health deficit model selecting items from the existing comprehensive geriatric assessment (CGA) questionnaire, could develop a clinically useful frailty index named the FI-CGA (10, 11). Other common frailty instruments have been proposed, such as the FRAIL scale, the Clinical Frailty Scale (CFS), and the Groningen Frailty Indicator (GFI) (3).

Several frailty guidelines or consensuses recommend frailty screening for older adults (3, 12–14), especially in primary care settings, using some commonly validated frailty assessment tools (Table 1). However, screening or evaluating frailty is not implemented in routine clinical practice (16). An important reason for this is that most frailty screening and assessment tools are based on specific questionnaires or physical examinations, which add to the clinical workload (17, 18). Furthermore, some frailty assessment tools consist of numerous multi-dimensional items and may require the guidance of geriatric specialists and training for assessors, which are the main obstacles to the conversion of frailty research to clinical practice (e.g., CGA, the “golden standard” to diagnose frailty, is complex and labor intensive and can't be readily available in day-to-day practice) (17).

**Table 1:** Common frailty assessment tools recommended in primary care (1, 3, 12–15).

**Tool**	**Components**	**Classification**
Frailty Index (FI)	Any 30 or more accumulated health deficits (variables) that increase in prevalence with age but do not plateau with age. Variables should be multidimensional, including functional status, laboratory abnormalities comorbidities, mood, cognition, and nutritional status.	Continuous score ranging from 0 to 1; > 0.25 is often selected to define frailty
Frailty phenotype	Five items: weight loss; slow walking speed; low grip strength; exhaustion; low physical activity	Frailty: ≥3 items; prefrailty: 1–2 items; robust: 0 items
FRAIL scale	Five items: fatigue; resistance (ability to climb up one flight of stairs); ambulation (ability to walk one block); illness (> 5 comorbidities); loss of weight (> 5%)	Frailty: ≥3 items; prefrailty: 1–2 items; robust: 0 items
Clinical Frailty Scale (CFS)	Visual chart for frailty with nine graded pictures, with corresponding explanation text.	Ranging from 1 (very fit) to 9 (terminally ill); Frailty: score ≥5
Study of Osteoporotic Fractures (SOF)	Three items: weight loss, exhaustion, unable to rise from a chair five times without using arms	Frailty: ≥2 items; prefrailty: 1 item; robust: 0 items
PRISMA-7	Seven self-reported items: age (>85 years), male, social support, and ADLs	Frailty: score ≥3
Tilburg Frailty Indicator (TFI)	Contains two parts: 10 questions on determinants of frailty and diseases (Part A) and 15 questions on components of frailty in three domains (physical, psychological, and social frailty) (Part B)	Frailty: score ≥5
Groningen Frailty Indicator (GFI)	Fifteen self-reported items in four domains: physical, cognitive, social, psychological	Frailty: score ≥4
Short Physical Performance Battery (SPPB)	Three measured items: gait speed, standing balance, and repeated chair stands	Each item scored from 0–4, maximum score of 12; Frailty: score ≤ 9
Timed Up and Go (TUG) test	The test measures the time taken to stand up from a chair, walk a 3-meter distance, turn, walk back and sit down.	A time of >10 s identifies patients at risk of frailty
Edmonton Frailty Scale (EFS)	Nine items: cognition, health (two items), hospitalization, social support, nutrition, mood, function, and continence	Frailty: score ≥7
Kihon Checklist	Twenty-five dichotomous items in seven categories: physical strength, nutrition, eating, socialization, memory, mood, and lifestyle; scoring as per the Frailty Index	Continuous score; suggested frailty cut-off score >0·25
SHARE Frailty Instrument (SHARE-FI)	Includes five variables: exhaustion, weight loss, weakness (as assessed by handgrip strength using a dynamometer), slowness, and low activity	Web-based calculator distinguishes three categories: non-frail, prefrail and frail
Gait speed	The patient is asked to walk from one place to another at the usual speed. Distance considered ranges from 2.4 to 6 m.	A walking speed of <0.8 m/s identifies patients at high risk of frailty.

Medical data naturally generated by routine work, such as symptoms, signs, diagnoses, abnormal laboratory tests, and lifestyle information, are stored in the EHRs (some of this information is formally coded) (19, 20). These data could be indicators of frailty assessment tools, especially the frailty index based on the cumulative deficit model, providing new resources and possibilities for frailty assessment (18, 20). Using frailty assessment tools based on EHRs (EHR-based frailty assessment tools) does not require additional resources and may bridge the gap between frailty research and clinical frailty assessment (17, 20). An integrative review in 2021 compared the EHR-based frailty assessment tools from different settings, especially the items and the mechanisms involved. It mentioned the advantages of primary care EHRs in constructing frailty assessment tools but failed to describe the application of relevant tools in detail (21). Similarly, some studies had pointed out that primary care EHRs are more comprehensive and representative compared with EHRs in hospitals (22, 23). However, there is currently a lack of a detailed summary of new studies focusing on frailty identification by primary care EHRs. This narrative review aims to describe the current status of identifying frail patients in primary care through EHRs, as well as challenges and future directions.

## EHR-Based Frailty Assessment Tools in Primary Care

### Electronic Frailty Index (eFI)

In 2013, Clegg et al. described the possibility and necessity of constructing frailty instruments based on the existing clinical data set in primary care (2). Applying the cumulative deficit frailty model as the theoretical framework (8), they developed and validated the eFI based on EHRs in ResearchOne and The Health Improvement Network (THIN) primary care databases in the UK. Thirty-six health deficits (Table 2) were used to calculate individuals' eFI scores, which defined the categories of fit, mild frailty, moderate frailty, and severe frailty (18) (Table 3). In the internal validation cohort (ResearchOne databases), including over 200,000 people aged 65–95, those with severe frailty (eFI scores > 0.36) had a 5-fold greater 1-year risk of death (the hazard ratio (HR) was 4.52), unplanned hospitalization (HR 4.73), and nursing home admission (HR 4.76) than the fit group (eFI scores 0–0.12) (17, 18). The external validation cohort (THIN databases) showed broadly similar predictive validity for mortality (for severe frailty, 1-year mortality *HR* 4.50) (18).

**Table 2:** List of 36 health deficits in the eFI by Clegg et al. (18).

**List of 36 health deficits**			
Activity limitation	Falls	Ischaemic heart disease	Respiratory disease
Anemia and haematinic deficiency	Foot problems	Memory and cognitive problems	Skin ulcer
Arthritis	Fragility fracture	Mobility and transfer problems	Sleep disturbance
Atrial fibrillation	Hearing impairment	Osteoporosis	Social vulnerability
Cerebrovascular disease	Heart failure	Parkinsonism and tremor	Thyroid disease
Chronic kidney disease	Heart valve disease	Peptic ulcer	Urinary incontinence
Diabetes	Housebound	Peripheral vascular disease	Urinary system disease
Dizziness	Hypertension	Polypharmacy	Visual impairment
Dyspnoea	Hypotension/syncope	Requirement for care	Weight loss and anorexia

**Table 3:** Characteristics of each EHR-based frailty assessment tool in primary care.

**Tool**	**Author**	**Year**	**Country/region**	**Database** **source**	**Number of frailty items**	**Components**	**Classification**	**Range of score**	**Outcome**
eFI	Clegg et al.	2016	United Kingdom	The ResearchOne and The Health Improvement Network (THIN) databases	36	Clinical and administrative data from EHR, including symptoms, signs, laboratory test results, diseases, disabilities, and information about social circumstances	Mild frailty 0.13–0.24; Moderate frailty 0.25–0.36; Severe frailty >0.36	0–1	Mortality; Hospitalization; ED visit; Nursing home admission; falls; Fracture; Stroke; bleeding; Cost et al.
Drubbel-FI	Drubbel et al.	2013	Netherlands	Administrative routine healthcare data from GPs EMR in Utrecht, Netherlands	36; 50	International Primary Care Classification codes (including general complaints, symptoms, functional impairments, diseases, social, psychological, cognitive impairment); polypharmacy (≥5 medications)	For adverse health outcomes: Low risk: 0.00–0.03; Intermediate risk: 0.04–0.13; High risk ≥0.14; or frailty≥0.08; or frailty≥ 0.2	0–1	Mortality; Nursing home admission; Oral health; ED and after-hours GP surgery visits et al.
CAN	Ruiz et al.	2018	United States	VA Computerized Patient Record System EHR, USA	65	Medical conditions, number of diagnoses, vital signs, medications, laboratory tests, use of care coordination resources, and overall VA healthcare utilization (6 categories including demographics, chronic illness, utilization, vital signs, pharmacy, and Interactions)	Compared with a 40-item CGA-FI: the score of 55: sensitivity 91.67%, specificity 40.32%; the score of 95: sensitivity 43.33%, specificity 88.81%	Displayed as a percentile: low risk, 0 to high-risk, 99	Mortality; Hospitalization et al.
VA-FI	Orkaby et al.	2019	United States	National VA administrative and Medicare and Medicaid data, USA	31	Morbidity (arthritis, diabetes et al.), functional status (codes for debility and durable medical equipment), cognition and mood dementia and depression, sensory impairment (hearing or visual impairment et al.), and other geriatric syndromes (incontinence et al.)	Non-frail 0–0.10; Prefrail 0.11–0.20; Frailty>0.2; Mild frailty 0.21–0.30; Moderate frailty 0.31–0.40; Severe frailty≥ 0.41	0–1	Mortality; Hospitalization et al.
Adapted eFI	Pajewski et al.	2019	United States	Medicare Accountable Care Organization (ACO) EHR data, USA	54	Vital signs, diagnosis code, laboratory, medication, and Medicare Annual Wellness Visit (AWV) (including functional data)	Non-frail ≤ 0.1; Prefrail >0.10; Frailty >0.21	0–1	Mortality; Treatment complications; Healthcare encounters; ED visits; Injurious falls; Hospitalization; Readmission et al.

Several studies have investigated the psychometric properties of the eFI. Hollinghurst et al. used the Secure Anonymised Information Linkage (SAIL) Databank, including 469,000 people aged 65–95, to further validate the eFI (24). *HR* trends for mortality consist of the initial findings from the previous two databases, which showed robust predictive validity of the eFI (24). Three British studies compared the eFI with several frailty tools to investigate its construct validity for frailty identification (25–27). One pilot study showed that those who were referred to the CGA clinic had higher mean eFI scores (0.33 vs. 0.23) (25). Another study including 353 older adults (≥75) showed that the eFI had a strong Spearman correlation coefficient with the research standard FI and Edmonton Frail Scale, as well as a moderate correlation with the CFS and phenotype model (26). However, in a similar study including 265 older people (mean 85.6), the eFI may have overestimated frailty status in comparison with CFS [odds ratio (OR) is 5.43] (27). A Canadian study showed that the manually calculated eFI from primary care EHRs in Canada had a strong correlation with the FI-CGA (Pearson correlation coefficient is 0.72) (28). In terms of feasibility and acceptability, the manually calculated eFI only needed patients' EHRs and 10–20 min to conduct, which is less intrusive than the PRISMA-7 or 4-m walk test and accepted by clinicians such as those in the previous British pilot study (25). Similarly, it is feasible to extract an eFI with a high discriminative ability (the area under the curve (*AUC*) is 0.9; vs. frailty phenotype) from routinely collected Australian primary care data, although 15% of records were difficult to extract (29). In general, the eFI is a valid, practical, sensitive, and time-efficient [in 5 min (25)] screening tool with which to identify frailty.

The eFI was soon recommended in the GP contract in England (in the new 2017/18 quality and outcomes framework, which is a fundamental part of the general medical services contract) (30). GPs can calculate eFI scores easily by using the existing software to identify at-risk patients in daily clinical practice under the guidance of webinar recordings (Watch the video for the operation of the eFI calculator on http://help.visionhealth.co.uk/DLM550/Visionplus/index.htm#77063) and other online resources from the website of NHS and then make a diagnosis by reviewing an individual's complete clinical history (30–32).

### The Drubbel-Frailty Index

In 2013, Drubbel et al. constructed a 36-deficit frailty index using the International Primary Care Classification (ICPC) codes and a polypharmacy deficit from the primary care EHR database in Utrecht, Netherlands (33), marking the formation of the first EHR-based frailty assessment tool (also called electronic Frailty Index-Utrecht, and eFI-U). The study, which included 1,679 older patients demonstrated that the Drubbel-FI had moderate predictive power for adverse outcomes (c-statistic was 0.702) (33). Further study found that the Drubbel-FI had moderate overlap with the Groningen Frailty Indicator (GFI) (Pearson correlation coefficient is 0.544) and may cover different aspects or stages of frailty (34). To date, the Drubbel-FI has only been used as a frailty screening tool in some studies in the Netherlands, with 36 or 50 deficits and different cut-off values in different studies (35–37). It may take time for this tool to be put into clinical practice in primary care.

### Care Assessment Need Score

The Care Assessment Need score (CAN) was initially developed by the Department of Veterans Affairs (VA) as a risk prediction tool for hospitalization and mortality in veteran populations in the US (38). The Veterans Health Administration, which is part of the VA, is a national integrated healthcare system that captures and stores claims and healthcare data in a centralized database (39). The CAN score can be automatically generated from EHRs in the VA primary care database (38). Due to the similarity in data elements and calculating methods between the CAN model and deficit accumulation model, Ruiz et al. validated the CAN score as a frailty identification tool in 2018 (38, 40). The CAN score was significantly increased from the robust to prefrail and frail groups and showed a moderate association with the FRAIL scale (Spearman correlation coefficient is 0.437) (38). Compared with the 40-item CGA-FI, a study including 184 patients over age 65 demonstrated that the CAN score had acceptable diagnostic accuracy (*AUC* 0.736) and had high sensitivity and negative predictive value at the 55th percentile of cut-off scores (40). In short, the CAN score may be a useful tool for frailty identification in primary care but is only available in the US VA system.

### Veterans Affairs Frailty Index

Another computer-generated VA instrument, the Veterans Affairs Frailty Index (VA-FI) was constructed with 31 deficits based on claims data (including diagnostic and procedure codes) from national VA administrative, Medicare, and Medicaid data in 2019 (41). A study including nearly 3 million veterans who regularly visited VA clinics revealed that the 2-year *HRs* of mortality for prefrail, mildly frail, moderately frail, and severely frail patients were 1.51, 2.36, 3.68, and 6.62, respectively (41). The original deficits of VA-FI were mainly identified from the international classification of diseases, the ninth revision (ICD-9 codes) in claims data, and the updated VA-FI with ICD-10 codes (VA-FI-10) maintained content validity, stability, and predictive validity for mortality (42). To be precise, the VI-FI is a claims-based frailty assessment tool rather than an EHR-based frailty assessment tool such as the CAN score. Therefore, it may be applied to different medical systems with claim data (including ICD diagnoses, etc.) and be further implemented in larger populations.

### Adapted eFI in Medicare Accountable Care Organization

The adapted 54-item eFI was constructed by Pajewski et al. using data from the Medicare Accountable Care Organization (ACO) in the USA (ACO is an organization of clinically integrated health care providers who give coordinated high-quality care to their Medicare patients). It involved health deficits that were previously validated in the eFI (18) and FI-LAB (based on routine physical and laboratory tests) (43), such as diagnostic codes, laboratory measurements, and medications (except the functional deficits from healthcare center visit data) (44). In this study, which included 12,998 patients, the adapted eFI independently predicted mortality, hospitalisations, emergency department (ED) visits, and falls in primary care (44). It seems feasible to apply an EHR-based FI in the managed care population in the US. However, further extensive studies are needed to investigate the psychometric properties of the adapted eFI.

## Application of These Tools

### Screening Frailty at Population Levels

In theory, EHR-based frailty assessment tools can automatically generate frailty scores for any person with EHRs, enabling frailty screening at the population level. Both the eFI and VF-FI were implemented in large sample (200,000–3,000,000) population studies (18, 24, 41). Clegg et al. found estimates of prevalence for mild, moderate, and severe frailty of 35–37, 12–16, and 3–4%, respectively, in British older adults from the ResearchOne and THIN databases (18). Similar results were found in the Welsh population in the SAIL Database (24). Another British study, which included 2,177,656 participants, demonstrated that 10% of adults aged 50–64 and 43.7% of older adults were affected by frailty (eFI > 0.12) (45). For older veterans in the US, 28.3% were prefrail, 19.7% were mildly frail, 12.7% were moderately frail, and 14.3% were severely frail in 2012 (41). The abundance of cases in large population studies may offer immense research opportunities, such as assessing differences in the prevalence of frailty among different ethnic groups in multiethnic countries. A study including 13,510 older adults in London demonstrated that the overall prevalence of frailty (eFI sores > 0.24) was 18.1%, with the highest prevalence in the Bangladeshis population (32.9%) and the lowest in the black population (4.0%) (46). In contrast, those with a higher prevalence of frailty in the United States are African Americans and Hispanics in a sample of over 16,000 older US veterans (47).

### Monitoring Frailty Trends, Transitions, and Trajectories

Patients with regular follow-up in primary care have continuous EHRs that can repeatedly generate frailty scores at different time points and provide information on changes in frailty with aging (5). This may aid research that previously seem difficult, such as investigating changes in frailty prevalence and frailty incidence, frailty transition, and frailty trajectories. The prevalence of frailty in older US veterans has increased over time (41). The overall rate rose from 32 to 47% in 2002–2012, and the proportion of veterans diagnosed with severe frailty increased from 4.3 to 14.1% (41). Consistent with the high prevalence, a high incidence of frailty was also found in older veterans (47). Frailty transition periods reveal a shorter time between frailty categories (47). The Welsh study described a median transition time from fit to mild, mild to moderate, and moderate to severe frailty of 2,165, 1,155, and 898 days, respectively, implying that frailty may accelerate in later stages (24).

As frailty is a dynamic and reversible state, understanding frailty trajectories within individuals and across the population is necessary. A British case-control study with a sample of 25,000 patients (≥75) investigated frailty trajectories by eFI scores and their impact on mortality (within 12 months) (48). Three different frailty trajectories were identified, followed by stable, moderately increasing, and rapidly rising frailty trajectories (48). A rapid rising frailty trajectory (starting at 0.21 at baseline, with monthly eFI score increases of 0.022 until the curve flattens) was associated with a 180% increase in mortality (*OR* 2.84) (48). A similar study, which included 214,250 US veterans with annual VA-FI scores in the 5 years before death (79.4 mean age at death), identified nine frailty trajectories (including 2 stable, 3 gradually increasing, 3 rapidly increasing, and 1 recovering trajectory) (49). Monitoring frailty trends, transitions, and trajectories may help physicians identify suitable candidates for prevention, treatment, or palliative care at the appropriate time and further study the etiology of frailty, such as the association between some common frailty risk factors (such as area-level deprivation) (50) and frailty trajectories.

### Stratified Management of Chronic Disease(s)

Although frailty and chronic diseases are two different concepts, they are closely related (51–54). According to the deficit accumulation model, chronic disease(s), multimorbidity, or comorbidity are important components of frailty (51, 55). Moreover, the eFI was recommended in the 2016 UK National Institute for Health and Care Excellence multimorbidity guidelines as one of the validated tools to identify adults with multimorbidity who are at risk of adverse events (56).

Consistent with previous studies on frailty and chronic disease(s), studies using EHR-based frailty assessment tools in primary care also observed a positive association between frailty and chronic disease. Among 3 million older US veterans, frailty evaluated by VI-FI was associated with an increased risk of cardiovascular disease (CVD) mortality at all levels of frailty and an increased risk of myocardial infarction and stroke (57). Similarly, frailty was found to be prevalent in patients with atrial fibrillation (AF) (58), hypertension (59, 60), and multiple myeloma (61, 62), and higher frailty levels were associated with increased mortality in those patients (58–62). In addition, frailty, rather than comorbidities, was one of the main predictors of all-cause admissions in patients with heart failure in England (63).

Previous studies have suggested that frailty needs to be considered in the treatment of chronic disease(s) based on individuals' frailty status (64). It is observed that frailty appears to influence whether older patients with chronic disease(s) choose to have surgeries. A recent British study, which included 28,025 individuals with hip osteoarthritis in primary care, showed that increased pre-existing multimorbidity (especially defined by the eFI) was associated with a decreased likelihood of undergoing total hip arthroplasty (65). However, several real-world studies using EHR-based frailty assessment tools showed that the impact of frailty was not considered in drug treatment for patients with chronic diseases, leading to inappropriate pharmacological treatment in the most vulnerable patients (58, 59, 66). In a sample of 244,328 community-dwelling Dutch people aged 70 and older, lipid-lowering drug prescriptions decreased with age but increased with higher frailty levels, which may imply potential overtreatment (66). Two British studies using the eFI may provide some insights into antihypertensive treatment among older adults with hypertension (59, 60). A study including over 140,000 hypertension patients aged 80 and older in England showed that the mortality rates were greatest at SBP <110 mm Hg (59). Another study with 415,980 patients aged above 75 found that the risk of all-cause mortality was lower when blood pressure was 150–159 mmHg than 130–139 mmHg in those severely frail individuals aged 75–84 years (60). These results suggest that hypertensive patients with automatically generated high frailty scores should be monitored closely as their antihypertensive therapy needs to be used with caution to avoid overtreatment. A more complicated situation is reflected in the management of stroke prevention in frail patients with AF who are taking oral anticoagulants (OACs). One study, which included a half-million older adults in England showed that among patients with AF and high stroke risk, OACs prescription was more common in those with frailty (58). Considering frail patients' higher risk of death, gastrointestinal bleeding, and falls, stratified stroke prevention based on frailty scores may benefit the most vulnerable patients with AF (58). On the other hand, frailty assessment may also be beneficial for patients who are at high risk for developing frailty and taking strong anticholinergic medications (67).

In summary, it is feasible to evaluate frailty in populations with chronic diseases by using EHR-based frailty assessment tools, as demonstrated in these primary care studies (See the brief list of the studies involved in Table 4). The research results further suggest the necessity of assessing frailty, and the automatically generated frailty scores can provide a reference for clinical decision-making [e.g., as a modifier of the risk-benefit ratio in pharmacological treatment (64)] and as a signal to refer a patient for a comprehensive geriatric assessment that will help develop an individualized treatment plan that will consider his or her chronic disease(s).

**Table 4:** Studies involving chronic diseases and EHR-based frailty assessment tools in primary care.

**Author**	**year**	**Chronic diseases**	**Study design**	**Population**	**Tools^**a**^**	**Main outcomes**
Shrauner et al. (57)	2021	Cardiovascular disease; myocardial infarction stroke	Cohort study	3,068,439 US Veterans aged ≥65	VI-FI	Frailty was associated with an increased risk of cardiovascular mortality at every level of frailty; Frailty was also associated with an increased risk of myocardial infarction and stroke.
Wilkinson et al. (58)	2021	Atrial fibrillation (AF)	Cohort study	536,955 patients aged ≥65	eFI	AF prevalence and mean CHA_2_DS_2_-Vasc for those with AF increased with increasing eFI category; For AF with CHA_2_DS_2_-Vasc ≥2, OAC prescription was higher for mild (53.2%), moderate (55.6%), and severe (53.4%) eFI categories than fit (41.7%); In those with AF and eligible for OAC, frailty was associated with an increased risk of death (HR for severe frailty compared with fit 4.09, 95%CI 3.43-4.89), gastrointestinal bleeding (2.17, 1.45-3.25), falls (8.03, 4.60-14.03) and, among women, stroke (3.63, 1.10–12.02).
Bottle et al. (63)	2019	Heart failure (HF)	Cohort study	6,360 patients diagnosed with HF	eFI	The main predictors of all-cause admission were age, co-morbidity, frailty, prior admission, not being on a beta-blocker, low haematocrit, and living alone; Frailty effects were largest in patients aged under 85.
Ravindrarajah et al. (59)	2017	Hypertension	Cohort study	144 403 participants aged ≥80	eFI	Mortality rates increased with frailty level and were greatest at SBP <110 mmHg; In fit women, mortality was 7.7 per 100 person-years at SBP 120 to 139 mmHg, 15.2 at SBP 110 to 119 mmHg, and 22.7 at SBP <110 mm Hg; For women with severe frailty, rates were 16.8, 25.2, and 39.6, respectively; SBP trajectories showed an accelerated decline in the last 2 years of life; The relative odds of SBP <120 mmHg were higher in the last 3 months of life than 5 years previously in both treated (*OR* 6.06; 95% CI 5.40-6.81) and untreated (6.31; 5.30–7.52) patients.
Masoli et al. (60)	2021	Hypertension	Prospective cohort study	415,980 primary care patients aged ≥75	eFI	Associations with mortality varied between non-frail <85 and frail 75–84-year-olds and all above 85 years; SBPs above the 130–139-mmHg reference was associated with lower mortality risk, particularly in moderate to severe frailty or above 85 years; SBP <130 mmHg and DBP <80 mmHg were consistently associated with excess mortality, independent of BP trajectory toward the end of life.
DuMontier et al. (61)	2021	Multiple myeloma (MM)	Retrospective cohort study	4,924 transplant-ineligible veterans aged ≥ 65 with MM	VI-FI	Survival and time to hospitalization decreased with increasing VA-FI severity; The VA-FI predicted mortality and hospitalisations.
Ferguson et al. (65)	2021	Osteoarthritis (OA)	Cohort study	28,025 patients aged over 65 years with hip OA	eFI	Increased multimorbidity was associated with a decreased likelihood of undergoing THA, irrespective of the method of assessing multimorbidity although the impact varied by approach.

^a^*EHR-based frailty assessment tools in primary care*.

### Predicting Patients' Specific Healthcare Needs

The novel frailty assessment tools may also help to identify patients with high medical needs and the generated frailty score could be an indicator of healthcare usage. A British cohort study, which enrolled 22,859 older adults with eFI scores, showed that an increased level of frailty is associated with increased acute hospital admission, more community referrals, and more requirements for care plans. Similarly, an analysis of linked routine primary care records from approximately 100,000 participants aged 65–95 showed that frailty was associated with increased hospital admissions, increased GP consultations, and longer inpatient stays (68). The total additional costs for older people with frailty were approximately £6 billion per year across the UK (68). The results imply that eFI scores could be an indicator of community service usage and might aid in the allocation of healthcare resources. In addition, EHR-based frailty assessment tools could also identify patients with other specific medical needs. A study involving 265,195 people over the age of 80 found that among women, the incidence of each fracture type was high and increased with the frailty category (69). Strategies for fracture prevention should target older women with frailty (69). Similarly, frailty (by the adapted eFI) was associated with greater post-acute care needs, higher 30-day readmission rates, and higher all-cause mortality within 6 months for non-urgent surgery (70). Furthermore, adapted eFI scores >0.32 may identify patients most likely to benefit from in-home pharmacist medication reviews, as shown in a small sample study (71). At last, the eFI has also been attempted to predict in-patient mortality after hospitalization or ICU admission for critically ill community-dwelling patients, but maybe less predictive value than the hospital frailty risk score (HRFS, constructed from hospital data, mainly ICD codes) (72, 73). Similarly, building a new frailty tool using hospital discharge diagnostic data (ICD-10 codes) may address quick frailty assessment in patients returning from hospital to the community (74). Further application research on such tools may promote the development of evidence-based healthcare services targeting frailty to prevent adverse events and reduce health costs.

## Disadvantages and Challenges

Although automatically generated frailty scores are a time-efficient, low-cost process that takes frailty assessment closer to clinical practice, the existing deficiencies may present challenges to the application of EHR-based frailty assessment tools in different health systems around the world. First, variables or deficits of frailty are normally recorded as unstructured data that are difficult to extract, and some deficits are not fully available (29) in primary care EHRs. Bery et al. analyzed 135 frailty assessment tools (containing 593 frailty variables) published between 2011 and 2018 and pointed out that only 22 frailty tools may rely solely on EHRs and administrative claims data (75). Similarly, Sultana et al. found that clinical assessments of cognition (Mini-Mental State Examination score), mobility, and cachexia were not routinely recorded (<3% among 314,191 elderly persons) in the Dutch Integrated Primary Care Information (IPCI) database (36). Deficits such as functional limitations or mobility (33, 62, 76), social determinants of health, and health attitudes (33, 75) are not commonly recorded in primary care records, and symptoms and signs are not well-recorded (75), which may lead to a narrower FI score range. that underestimates frailty (33). In contrast, temporary diseases or conditions that have been cured may be recorded as deficits and may also lead to a higher estimate of frailty (27). Second, EHR-based frailty assessment tools also face the challenges of variation in EHR software and EHR data quality in different organizations (77) due to the lack of standards in how each EHR database records, processes, and stores data. Further training of eFI on health providers, validation of data quality, and software improvements are also needed to support better use of these tools and reduce the variability of data across facilities. Furthermore, tools like eFI are highly dependent on a well-established and homogeneous primary health service system. In other words, some countries may not be in the position to apply those tools, and only the eFI is currently in clinical use. However, the current diagnosis of frailty has been highly survey-based rather than using the eFI even in the UK. A study found most patients did not have eFI scores when coded as frail (only diagnosed by clinicians using tools such as the PRISMA-7 or the GFI) (77). Therefore, more research is needed to improve EHR-based frailty tools and EHR systems and provide corresponding support measures to promote the application of these tools.

## New Trends

### Applying Artificial Intelligence (AI)

Technological advances such as AI algorithms can make full use of a wide breadth of data derived from EHRs for the detection of diseases or conditions and may have potential value in screening frailty. Recent studies of patients in residential care facilities in Australia have shown that artificial intelligence technology may be a feasible approach for evaluating frailty (sensitivity 0.978, specificity 0.891, compared with the eFI) (78). AI is also credited with improving the predictive performance of a modified frailty index based on Hong Kong hospital data (79). However, in the primary care setting, Williamson et al. used data from the Canadian Primary Care Sentinel Surveillance Network (*n* = 875 adults aged over 65 years) to develop the EHR-based frailty definition by machine learning methods and showed that it had the poor predictive ability (sensitivity 28%, specificity 94%) compared with the CFS (80). However, another study based on the same database with a larger sample improved the predictive ability of this tool by using the XGBoost model (stands for eXtreme Gradient Boosting, is a gradient-boosted decision tree machine learning algorithm) and changing the decision threshold (sensitivity 78.14%, specificity 74.41%) (81). In addition, machine learning, such as the natural language processing algorithm, may help extract frailty variables from unstructured data in EHRs (82) and make full use of EHRs in primary care. Future research is required to better understand the use of AI techniques to support frailty identification within primary care.

### Developing New Frailty Indicators in EHRs

As mentioned, several important deficits may not be available in EHRs in primary care, while some new variables may be complements and even indicators that could independently identify frailty. A British study including 154 patients over 65 showed that home visits, although not commonly coded in EHRs, could be a good frailty screening tool (sensitivity 87.23%, specificity 61.68%) compared with clinical diagnosis. Similarly, a sample of 159,325 patients from the British EHRs database showed that inflammatory markers [such as C reactive protein (CRP)] are strong predictors of all-cause mortality in primary care, with a comparable C-statistic (maximum value was 0.89 when containing age, sex, and CRP) to several previously developed frailty indices. In addition, EHR-based frailty case-finding may also rely on the innovation of software and the redesigned EHR system. A recent study, using reprogrammed primary care IT in the UK, created the Pathfields Tool that incorporated suspected frail patients (e.g., patients over age 90 with diagnoses of dementia and/or severe frailty by eFI or home visit) into the Pathfields High-Risk Cohort (83). Compared with an eFI score of more than 0.24, the Pathfields Tool identified more patients with previously undiagnosed frailty (confirmed by the CFS scale) (83). Another study used the openEHR framework to represent frailty in an aging population, which may help further the development of aging population-oriented systems (84).

### Promoting Changes in Medical Service Patterns

As mentioned, in countries where EHR-based frailty assessment tools already exist in primary care, their widespread use in clinical practice can capture and monitor population-level frailty levels, distribution, and trajectories and better allocate limited public health resources to the most vulnerable older adults. This may help bridge the gap between population health and public health services for older adults (75). Population-based health management may also require a more proactive integrated medical service pattern, and a study in the Netherlands provides a good example. This Dutch cluster-randomized controlled trial included 7,638 participants from 39 general practices with a follow-up of 12 months. Both interventions, using an electronic frailty screening tool plus standard GP care or the tool plus a nurse-led care programme, showed high probabilities of being cost-effective compared with usual care (37). It is also worth noting that Kaiser Permanente (KP), like the previously mentioned ACO, is another example of integrated care. It has numerous primary care settings and hospitals and generates enormous EHRs stored in KP HealthConnect (85, 86). These integrated EHRs are of enormous value to the electronic frailty assessment research, such as a recent study comparing the electronic frailty tools for the prediction of adverse outcomes of abdominal surgery using EHRs from KP (including a comparison of HFRS and eFI) (87). In addition, the KP pyramid, a population-based chronic care model, may inspire the application of tools like eFI in chronic disease risk stratification management (86, 88).

In the meantime, the assessment of frailty is necessary for specific groups, such as patients with chronic disease, which is underrepresented in current guidelines (63, 75). Designing different frailty assessment tools for specific groups based on records appears equally feasible without additional resources and may more accurately assess their risk (75). Similarly, the impact of chronic diseases or conditions also needs to be considered in interventions for frailty. For example, a dietitian acting as a first contact practitioner for frail patients rather than a GP may be a cost-effective way to alter the nutritional status of frail patients (eFI 0.26–0.36) (89). Additionally, depression interacts with frailty (by the eFI) to further reduce the daily functioning of frail patients, suggesting that the clinical management of frailty should integrate physical and mental healthcare (90).

The development of EHR-based frailty assessment tools will also have additional implications for healthcare systems, especially in countries where primary care is underdeveloped. Although the advantages of these tools are obvious, in these countries, EHR-based frailty assessment studies may first be generated from secondary or tertiary hospital databases given the relatively poor quality of primary care EHRs. For example, the only two current studies of the eFI in China were all based on hospital data systems (91, 92). Additionally, they may be generated from EHRs for specific groups, such as VA-FI and CAN scores for veterans, from health check-up data, such as the Korean Frailty Index (THE frailty index) (93), or pure claim data, such as the Claims-based frailty index (94). However, due to the lack of continuity of care or care limited to a particular group, these EHR data may not enable the accurate and continuous assessment of frailty and/or reflect the frailty of the entire population. The urgency of the frailty screening of the population may prompt the development of primary care and the integration of primary care and hospital data or claim data (The KP HealthConnect might be a good example), which will ultimately bring healthcare reform.

## Conclusion

The EHR-based frailty assessment tools do not require additional work or resources. In addition to accurately predicting adverse events, they can also achieve novel scientific and clinical uses, especially providing a reference for population health management and health resource allocation. However, only the eFI has been put into use in clinical practice in the UK thus far, and other tools need more validation. More importantly, challenges such as the poor quality of some EHRs and differences between EHR systems should be addressed urgently. Some new technologies, such as AI, and the development of new frailty indicators and frailty-related health systems may bring solutions and changes, while further research is needed.

Research on EHR-based frailty assessment tools in primary care is still in its infancy globally. Our assumptions and conjectures may not be fully objective because selection and evaluation biases are not known in this narrative review. However, given the advantages of primary care in the screening and management of frail patients, we consider more countries need to develop and validate frailty assessment tools based on their primary care EHRs to better address the public health challenges posed by an increasing aged population.

## Author Contributions

JL and XL conceived the review. JL drafted/wrote the manuscript. CZ, JS, QZ, and XL revised and edited all the version of the manuscript. YY and XF revised the sections. All authors contributed to manuscript revision and approved the submitted version.

## Funding

This research was supported by National Clinical Research Center for Geriatrics, West China Hospital, Sichuan University (No. Z20191009), 1·3·5 project for disciplines of excellence–Clinical Research Incubation Project, West China Hospital, Sichuan University (2018HXFH005), Science and Technology Department of Sichuan Province (2020YFSY0293), Science and Technology Plan Project of Sichuan Province (No. 2021JDR0296), the Scientific Research Project of the Chengdu Health, Health Commission of Sichuan Province (ZH2020-104), and Family Planning Commission (No. 2021062).

## Conflict of Interest

The authors declare that the research was conducted in the absence of any commercial or financial relationships that could be construed as a potential conflict of interest.

## Publisher's Note

All claims expressed in this article are solely those of the authors and do not necessarily represent those of their affiliated organizations, or those of the publisher, the editors and the reviewers. Any product that may be evaluated in this article, or claim that may be made by its manufacturer, is not guaranteed or endorsed by the publisher.
